# The Design and Adaptive Control of a Parallel Chambered Pneumatic Muscle-Driven Soft Hand Robot for Grasping Rehabilitation

**DOI:** 10.3390/biomimetics9110706

**Published:** 2024-11-18

**Authors:** Zhixiong Zhou, Qingsong Ai, Mengnan Li, Wei Meng, Quan Liu, Sheng Quan Xie

**Affiliations:** 1 School of Information Engineering, Wuhan University of Technology, Wuhan 430070, China; zhixiongzhou9@whut.edu.cn (Z.Z.); qingsongai@whut.edu.cn (Q.A.); mengnanli@whut.edu.cn (M.L.); quanliu@whut.edu.cn (Q.L.); 2 School of Electronic and Electrical Engineering, University of Leeds, Leeds LS2 9JT, UK; s.q.xie@leeds.ac.uk

**Keywords:** pneumatic artificial muscle, neural network, position control, grasp training, soft hand exoskeleton

## Abstract

The widespread application of exoskeletons driven by soft actuators in motion assistance and medical rehabilitation has proven effective for patients who struggle with precise object grasping and suffer from insufficient hand strength due to strokes or other conditions. Repetitive passive flexion/extension exercises and active grasp training are known to aid in the restoration of motor nerve function. However, conventional pneumatic artificial muscles (PAMs) used for hand rehabilitation typically allow for bending in only one direction, thereby limiting multi-degree-of-freedom movements. Moreover, establishing precise models for PAMs is challenging, making accurate control difficult to achieve. To address these challenges, we explored the design and fabrication of a bidirectionally bending PAM. The design parameters were optimized based on actual rehabilitation needs and a finite element analysis. Additionally, a dynamic model for the PAM was established using elastic strain energy and the Lagrange equation. Building on this, an adaptive position control method employing a radial basis function neural network, optimized for parameters and hidden layer nodes, was developed to enhance the accuracy of these soft PAMs in assisting patients with hand grasping. Finally, a wearable soft hand rehabilitation exoskeleton was designed, offering two modes, passive training and active grasp, aimed at helping patients regain their grasp ability.

## 1. Introduction

There has been an increase in patients with hand dysfunction caused by diseases such as hemiplegia and strokes [1]. According to statistics, around 13.68 million people suffer new strokes globally each year, with about 70% of survivors experiencing some degree of upper limb and hand motor function impairment [2]. About 67% of stroke complication patients are still unable to use their hands even four years after onset. Key goals of hand rehabilitation include grasping objects, transferring, manipulating, coordinating finger movements, and maintaining flexibility [3]. Repetitive hand flexion and extension training is required to restore motor nerve function [4,5]. In clinical practice, rehabilitation is primarily conducted by physicians, and while effective, this approach has low efficiency. Consequently, researchers globally have extensively investigated the design and control of hand rehabilitation robots. Rigid structure hand exoskeletons [6,7], mainly driven by linkages, motors, and ropes, and underdriven structure hand rehabilitation robots [8] using spring drives offer precise control but have poor wearability. These rigid or semi-rigid devices can cause secondary injury during improper operation.

Soft robots driven by pneumatic artificial muscles (PAMs), made from soft materials such as silicone rubber and fabric, produce movements like bending, elongation, and twisting through changes in air pressure. This significantly enhances wearability and safety compared to rigid robots [9]. Soft rehabilitation robots offer unparalleled advantages in human–robot interaction, rehabilitation training, and assisted grasping.

Research has shown that PAM actuators, using pneumatic pressure as a power source, have many advantages. They are a central focus and challenge in the design and control of soft rehabilitation robots and represent an important trend for future development [10]. In recent years, soft PAMs that fit the body have been applied in soft rehabilitation exoskeletons. A typical bending PAM is made of silicone rubber airbags. Asymmetric airbags or limiting layers with fibers result in varying degrees of elongation on both sides of the PAM, enabling unidirectional bending [11]. Han et al. [12] designed a fiber-reinforced soft pneumatic bionic actuator that bends by utilizing the strain difference between the chamber and the limiting layer. Wang Tianyu et al. [13] from Shanghai Jiao Tong University proposed a pneumatic grid torsion actuator with a programmable grid shape design. Liu Chih-Hsing et al. [14] developed a soft pneumatic bending actuator using a grid-type structure. Their hand claw comprises three grid-type bending actuators connected in parallel by end actuators. Panagiotis P. et al. [15] utilized the enhancement effect of fiber threads in different directions to design a new bending PAM. Integrating bending PAMs with soft fabric gloves to create a hand rehabilitation exoskeleton minimizes damage during repetitive rehabilitation training. This approach is crucial for improving rehabilitation efficiency in clinical settings, enabling remote rehabilitation, and reducing the repetitive workload of physicians [16].

However, existing research on soft hand rehabilitation devices faces several problems, such as a lack of soft multi-degree-of-freedom PAMs and an inability to create lightweight PAMs that adapt to the natural range of motion of fingers of different lengths. Consequently, many scholars have turned their attention to designing novel cavity and braiding structures, optimizing PAM structural parameters, and modeling PAM kinematics through finite element simulation.

Due to the characteristics of soft materials, such as their nonlinearity and complex fiber-constrained structures, realizing precise position control of hand rehabilitation exoskeletons driven by PAMs is a significant research direction [17]. Many researchers use simple PID control methods, but the control accuracy and speed are insufficient [18]. Some researchers delve into the dynamic characteristics of PAM to establish its dynamic model [19]. For the modeling of soft PAM actuators, empirical model building, geometric model analysis, and finite element analysis methods are commonly used [20]. Wang Boran et al. [21] proposed a way to establish the relationship between the compilation angle and output force using finite element simulation to analyze the PAM. For the very complex nature of the dynamics of fluid soft actuators, Wang Tao et al. [22] used a second-order transfer function to describe the kinetic behavior of the motion from the driving air pressure to the bending angle of the soft actuator. Given the advantages of neural networks in handling nonlinear problems, applying neural networks to PAM control can effectively reduce tracking errors and achieve better position control and trajectory tracking. Elgeneidy. K et al. [22] proposed a purely data-driven PAM modeling method for controlling the bending angle of the PAM actuator, and a data-driven modeling technique using a regression analysis and artificial neural networks was used to derive an empirical model based on the generated experimental data.

In this paper, we design a soft wearable hand rehabilitation exoskeleton for stroke patients. We develop parallel chamber bidirectional bendable PAM actuators, analyze the static bending characteristics using a finite element analysis, and optimize their structural parameters. The reduced order forms of aerodynamic muscle strain energy, generalized force, kinetic energy, and gravitational potential energy were substituted into the Lagrangian equation to obtain the dynamic equation of PAM, and considering the need for precise position control of PAMs during rehabilitation grasp training, an optimized radial basis function neural network adaptive control algorithm (RBFNNO) based on parameter and hidden layer node optimization is designed. Finally, we design a hand rehabilitation exoskeleton that uses PAMs to drive the patient’s fingers for grasp training. The rest of the paper is organized as follows: Section 2 describes the design and fabrication of the parallel chamber PAMs and soft exoskeleton. Section 3 presents the PAM dynamic modeling and control methods. Section 4 details the hand rehabilitation experiments. Section 5 contains the discussion, followed by the conclusion in Section 6.

## 2. Bio-Inspired Mechanical Design

**Figure 1:**
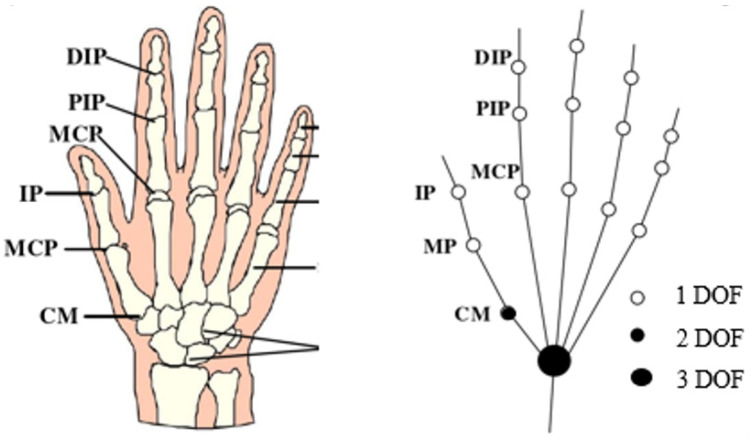
Hand bone structure and joint freedom.

Strokes, hand trauma, and nerve injuries lead to hand dysfunction, significantly impacting patients’ daily lives. The hand is a highly complex mechanism with over 20 degrees of freedom (DOFs). The DOFs of the hand joints are illustrated in Figure 1. In patients with hand dysfunction, muscle contraction and impaired joint movement reduce the DOFs and range of motion.

Traditional hand rehabilitation primarily relies on one-on-one repetitive training by physicians, but the low efficiency and insufficient training volume can result in suboptimal rehabilitation outcomes. Hand rehabilitation exoskeletons are clearly beneficial in reducing repetitive labor and enhancing rehabilitation quality. The hand exoskeleton is designed to drive the fingers to bend at specific angles and transmit appropriate forces to the fingers. To design a hand exoskeleton with a high fit, flexibility, and dexterity, the bidirectional bendable parallel chamber PAM is selected as the actuator to drive finger movement through muscle flexion.

**Figure 2:**
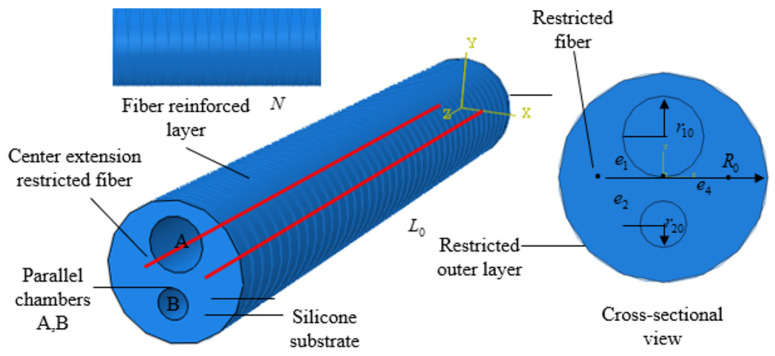
PAM structure design.

Considering the requirements for daily hand rehabilitation, this paper designed a parallel chamber PAM actuator by comparing the fiber-reinforced actuators developed by current researchers. Inspired by the motion of hand tendons, a parallel chamber structure was used in the design of the bending PAM to accommodate the needs of finger flexion and extension movements, as shown in Figure 2. The overall design of the actuator is cylindrical to ensure high power density and fast response. To meet the requirements for effective hand rehabilitation, the actual bending angle of the PAM should exceed 100°, and the fingertip force should be greater than 0.8 N.

The soft PAM consists of a silicone rubber shell and two independent asymmetric chambers. To limit the radial expansion and torsional bending of the PAM, Kevlar fiber lines are wrapped around the surface in a double-helix pattern, with the intersection of the helix ideally aligned along the axis of the chamber. The number of turns of the fiber lines affects the actual bending performance of the PAM. Too many turns increase stiffness, requiring higher inflation pressure to achieve the same bending effect, which reduces the PAM’s power density. Conversely, too few turns do not adequately constrain the PAM’s expansion, leading to excessive radial expansion that severely impacts bending performance.

Since the angles required for finger flexion and extension are different, Chambers A and B were designed with different radii. Chamber A is larger than Chamber B. When Chamber A is inflated, it produces significant bending to assist finger flexion, while Chamber B generates less bending to aid finger extension. Compared to single-chamber PAMs, our design of a double-chamber PAM enhances the extension function, providing better assistance for patients with muscle weakness.

In order to verify the actual bending effect, the simulation analysis of the PAM bending angle was carried out using ABAQUS finite element software (Version 6.14). The analysis of the soft body drive model using ABAQUS software mainly includes creating parts, setting material properties, and dividing the mesh, assembly, boundary conditions, and loads.

The Yeoh model can be simply used for the deformation behavior of nonlinear materials, and the model parameters can be well fitted by uniaxial tensile experiments, and the Yeoh model strain density function model is as follows.

(1) $$\;W=\sum_{i=1}^{N}C_{10}(I_{1}-3{)}^{i}+\sum_{k=1}^{N}\frac{1}{d_{k}}(J-1{)}^{2k}$$

(2) $$W=C_{10}(I_{1}-3)+C_{20}(I_{1}-3{)}^{2}$$

where *N*, $C_{i0}$, and $d_{k}$ are material constants determined by uniaxial tensile tests. The initial shear modulus $\mu =2C_{10}$.

**Figure 3:**
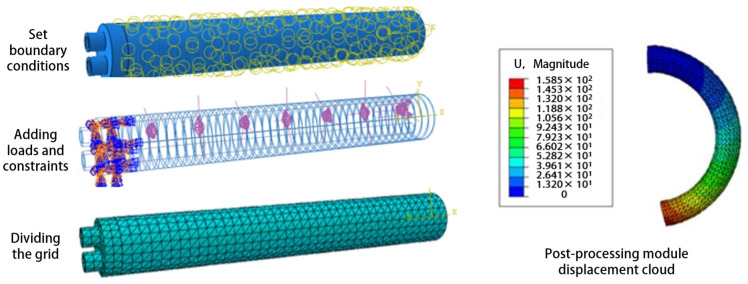
Finite element simulation boundary conditions and load settings.

The control variable method was used to simulate and compare various factors affecting the bending performance of PAMs in finite element simulations, as shown in Figure 3. This approach helps in selecting optimal parameters that meet finger rehabilitation requirements. The specific simulation results are detailed in Section 4.2. The optimized design parameters are listed in Table 1. A double-helix wire with a pitch of 3 mm was chosen for winding the PAM body, which has lengths of 100 mm and 120 mm. To prevent the fiber-wound wire from being exposed outside the silicone rubber housing, an additional 1 mm silicone rubber sleeve was added to cover the exterior of the PAM.

**Figure 4:**
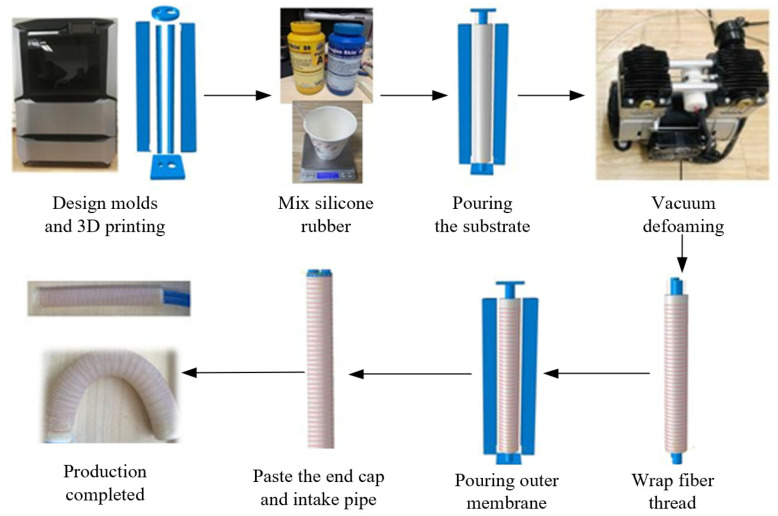
PAM manufacturing process.

We employ silicone casting to produce PAMs, utilizing a 1:1 mixture of Dragon-skin 30 silicone. The mold was designed using SOLIDWORKS based on specific design parameters and fabricated with a Stratasys F170 3D printer. The manufacturing process for the PAM, as illustrated in Figure 4, involves several key steps: mold design and production, mixing the silicone solution, pouring the matrix with cavities, attaching fiber threads, pouring the outer silicone, and assembling the end caps.

**Figure 5:**
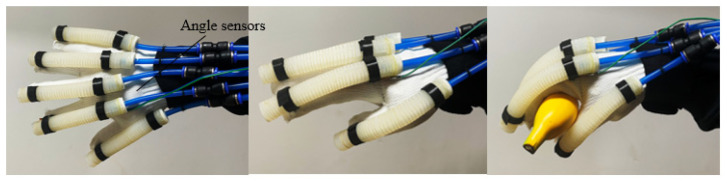
Soft hand exoskeleton.

Velcro is sewn onto the fabric glove. Velcro is used to secure the PAMs. The PAMs were secured to the distal interphalangeal (DIP) and metacarpophalangeal (MCP) joints with straps, fitting well with the patient’s fingers, as shown in Figure 5. To guarantee the durability of the PAM, each actuator is tested multiple times to confirm proper operation within a specific pressure range.

The bending angle range of the five fingers of the hand exoskeleton is determined by the PAMs. The thumb and little finger use 100 mm PAMs, while the index, middle, and ring fingers use 120 mm PAMs. As shown in Table 2, the five PAMs made for exoskeletons have different bending ranges. Patients with hand injuries cannot bend their fingers normally, making it difficult to reach the maximum bending angle of healthy fingers. It is also difficult to stretch after bending the fingers. The PAMs drive the movement of the patients’ fingers, enabling them to complete rehabilitation training.

The hand exoskeleton assisted the patient in performing various rehabilitation exercises. At different rehabilitation stages, the hand exoskeleton offers various modes to optimize rehabilitation outcomes, aiding in nerve recovery and enhancing hand movement abilities. In the early stage of rehabilitation, a passive training mode involving repetitive flexion and extension was designed for patients. The hand exoskeleton drove the patient’s fingers to complete these passive training tasks. As the patient’s hand strength and nervous system had recovered to some extent, an active grasping rehabilitation training mode based on the mapping of the healthy and affected side was implemented. The angular signal of the patient’s healthy hand during object grasping was collected by a motion capture system and used to control the exoskeleton to assist in grasping objects.

## 3. Methods

### 3.1. Pneumatic Muscle Dynamic Modeling

The PAM is primarily composed of silicone rubber and fiber threads. The relationship between the bending angle and the input air pressure can be established by analyzing a geometric model. The model is based on the following assumptions [23]: the silicone rubber material is incompressible when charged with air pressure, meaning the total volume of the silicone rubber remains constant; the actuator remains cylindrical during inflation and bending [24]; the actuator maintains a circular shape during bending and exhibits an overall constant curvature change; and the fiber helix restricts radial extension and is always in contact with the outer surface of the PAM.

**Figure 6:**
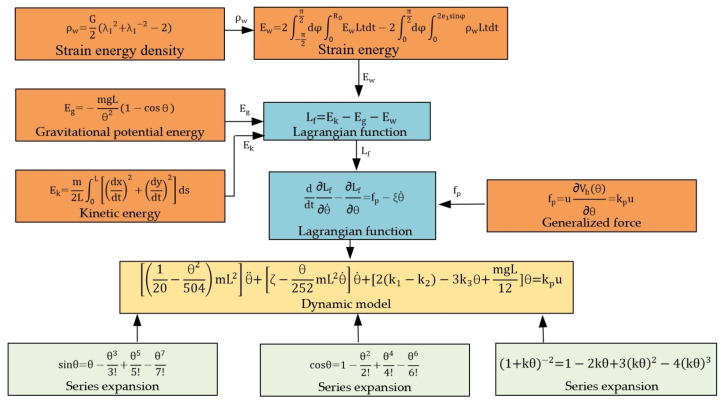
Block diagram of PAM dynamic derivation process.

The dynamic model of PAM is shown in Figure 6. The nonlinear terms in the strain energy density function and the sine and cosine terms in the robot dynamics are approximated using Taylor series. Lagrangian dynamics are an element of analytical mechanics that combines work, energy, and generalized coordinates to reformulate Newtonian mechanics. Unlike Newtonian mechanics, Lagrangian dynamics do not rely on a spatial coordinate system and do not require analysis of the binding forces within the system.

**Figure 7:**
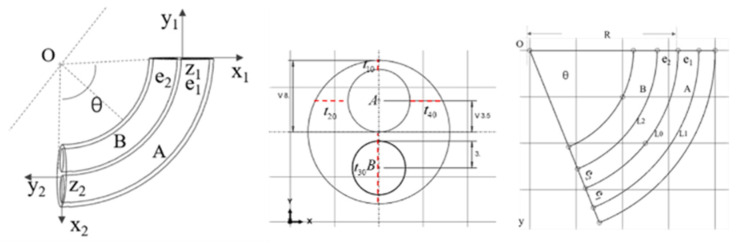
PAM geometry parameter model.

The cross-sectional and side views of the PAMs are shown in Figure 7. The bending radius R, eccentricity of cavity A $e_{1}$, eccentricity of cavity B $e_{1}$, and bending angle θ are selected to describe the bending degree of the actuator under varying air pressures. The radius $R_{0}$ represents the original radius of the pneumatic muscle, while $r_{10}$ and $r_{20}$ denote the original radii of cavities A and B, respectively. $L_{1}$ and $L_{2}$ represent the lengths of the central axes of cavities A and B after the pneumatic muscle inflates and deforms.

The strain energy density function of the Yeoh model is used to calculate the elastic strain energy, and the strain energy density function can be expressed as follows:

(3) $$W=C_{10}\left(\lambda_{1}^{2}+\lambda_{2}^{2}+\lambda_{3}^{2}-3\right)$$

In the above equation, $\lambda_{1}$, $\lambda_{2}$, and $\lambda_{3}$ are the axial, radial, and circumferential principal stretch ratios of the PAM, respectively. $C_{10}$ is the Yeoh model material parameter.

When a certain air pressure is filled in cavity A, the dimensional variation of the driver cavity in three directions—axial, radial, and circumferential—can be obtained according to the driver wall thickness parameters [25]:

(4) $$\left\{\begin{array}{c} \lambda_{1}=\frac{l_{1}}{L_{0}}\\ \lambda_{2}=\frac{r_{1}}{R_{0}}\\ \lambda_{3}=(\frac{t_{1}}{t_{10}}+\frac{t_{2}}{t_{20}}+\frac{t_{3}}{t_{30}}+\frac{t_{4}}{t_{40}})/4 \end{array}\right.$$

where $L_{0}$ represents the initial length of the central axis and $R_{0}$ denotes the initial radius of the PAM when the PAM is not deformed.

The thickness of the front and rear sides of cavity A are $t_{10}$ and $t_{30}$. The thickness of the left and right sides of cavity A are $t_{20}$ and $t_{40}$. After inflating cavity A, the thicknesses $t_{10}$/$t_{20}$/$t_{30}$/$t_{40}$ become $t_{1}$/$t_{2}$/$t_{3}$/$t_{4}$.

The PAM inflation deformation resembles a circular arc with the center of circle O, which can be obtained by geometric structure analysis:

(5) $$\left\{\begin{array}{c} t_{1}=R-e_{1}-r_{1}\\ t_{2}=\sqrt{R^{2}-{e_{1}}^{2}}-r_{1}\\ t_{3}=R+e_{1}-r_{1}\\ t_{4}=\sqrt{R^{2}-{e_{1}}^{2}}-r_{1} \end{array}\right.$$

By the binding of the radial fiber threads, the driver is barely deformed in the radial direction. It can be assumed that the radial stretch ratio $\lambda_{2}$ is 1. The axial stretch of the silicone rubber varies with the height in the cross section, and the axial stretch ratio $\lambda_{1}$ can be set as shown in the following equation, where β is the variation of the thickness along the bottom to the top.

(6) $$\lambda_{1}=1+\frac{\theta }{L}\beta$$

(7) $$\lambda_{3}={\lambda_{1}}^{-1}$$

The strain energy density is therefore rewritten as the following equation, where G is the shear modulus of the silicone rubber material.

(8) $$\rho_{w}=\frac{G}{2}\left({\lambda_{1}}^{2}+{\lambda_{1}}^{-2}-2\right)$$

The strain energy $E_{w}$ is expressed as integrating the entire silicone rubber, ignoring the uninflated cavity B.

(9) $$E_{w}=2\int_{-\frac{\pi }{2}}^{\frac{\pi }{2}}d\varphi \int_{0}^{R_{0}}E_{w}Ltdt-2\int_{0}^{\frac{\pi }{2}}d\varphi \int_{0}^{2e_{1}sin\varphi }E_{w}Ltdt$$

The gravitational potential energy $E_{g}$ is mainly determined by its average relative height in the vertical direction, and the gravitational potential energy can be expressed as follows:

(10) $$E_{g}=-mg\frac{\int_{0}^{\theta }R\sin\psi d\psi }{\theta }=-\frac{mgL}{\theta^{2}}\left(1-\cos\theta \right)$$

where m is the PAM mass and g is the gravitational acceleration. The analysis of kinetic energy reduces the drive to a curve, and the coordinates of each point on the drive can be expressed in the following equations:

(11) $$x(s)=\frac{L}{\theta }(1-\cos\frac{s\theta }{L}))$$

(12) $$y\left(s\right)=\frac{L}{\theta }\sin\frac{s\theta }{L}$$

where s is the arc length between the point on the PAM and the origin.

The total kinetic energy $E_{k}$ can be calculated by the following equation:

(13) $$E_{k}=\frac{m}{2L}\int_{0}^{L}\left[{\left(\frac{dx}{dt}\right)}^{2}+{\left(\frac{dy}{dt}\right)}^{2}\right]ds=mL^{2}\theta^{2}\left[\frac{1}{{6\theta }^{2}}+\frac{1}{\theta^{4}}\left(1+\cos\theta \right)-\frac{2}{\theta^{5}}\sin\theta \right]$$

According to the principle of virtual work, the generalized force caused by the inflation pressure can be expressed as follows:

(14) $$f_{p}=u\frac{\partial V_{h}\left(\theta \right)}{\partial \theta }=\pi \left[2r_{1}\left(L_{0}+e_{1}\theta \right)\cdot \frac{dr_{1}}{d\theta }+r_{1}^{2}\cdot e_{1}\right]u=k_{p}u$$

where u is the air pressure, $V_{h}\left(\theta \right)$ is the volume of the cavity A after inflation and deformation, and $k_{p}$ is the coefficient used to simplify the expression.

Based on the potential and kinetic energies calculated above, the Lagrangian function is as follows:

(15) $$L_{f}=E_{k}-E_{g}-E_{w}$$

The Lagrangian kinetic equations are as follows:

(16) $$\frac{d}{dt}\frac{\partial L_{f}}{\partial \dot{\theta }}-\frac{\partial L_{f}}{\partial \theta }=f_{p}-\xi \dot{\theta }$$

where ξ is the coefficient used to account for the damping characteristics and viscous resistance.

Using series expansion ${\left(1+k\theta \right)}^{-2}=1-2k\theta +3{\left(k\theta \right)}^{2}-4{\left(k\theta \right)}^{3}$ for Equation (8), we can obtain the following:

(17) $$\rho_{w}=\frac{G}{2}[1+4\frac{\theta^{2}}{L^{2}}\beta^{2}-4\frac{\theta^{3}}{L^{3}}\beta^{3}]$$

So, Equation (9) can be simplified as

(18) $$\begin{array}{c} E_{w}=G\int_{-\frac{\pi }{2}}^{\frac{\pi }{2}}d\varphi \int_{0}^{R_{0}}[1+4\frac{\theta^{2}}{L}t^{3}{sin}^{2}t-4\frac{\theta^{3}}{L^{2}}t^{4}{sin}^{3}t]dt\\ \;\;\;\;\;\;\;-\int_{0}^{\frac{\pi }{2}}d\varphi \int_{0}^{2e_{1}sin\varphi }\int_{0}^{R_{0}}[L+4\frac{\theta^{2}}{L}t^{3}{sin}^{2}\varphi -4\frac{\theta^{3}}{L^{2}}t^{4}{sin}^{3}\varphi ]dt=k_{1}\theta^{2}-k_{2}\theta^{2}-k_{3}\theta^{3} \end{array}$$

The sine and cosine functions in Equations (10) and (13) use the series expansion and ignore the terms above the sixth order in the cosine function and above the seventh order in the sine function as follows:

(19) $$E_{g}=mgL\left(\frac{\theta^{2}}{24}-\frac{1}{2}\right)$$

(20) $$E_{k}=\left(\frac{1}{40}-\frac{\theta^{2}}{1008}\right)mL^{2}{\dot{\theta }}^{2}$$

By substituting Equations (14) and (18)–(20) into Equation (16), the dynamic model can be obtained as follows:

(21) $$\left[\left(\frac{1}{20}-\frac{\theta^{2}}{504}\right)mL^{2}\right]\ddot{\theta }+\left[\zeta -\frac{\theta }{252}mL^{2}\dot{\theta }\right]\dot{\theta }+\left[2\left(k_{1}-k_{2}\right)-3k_{3}\theta +\frac{mgL}{12}\right]\theta =k_{p}u$$

When the derivative term is neglected, the equation simplifies to a static model.

### 3.2. RBFNN Adaptive Control

#### 3.2.1. Optimization Methods for RBFNN Nodes and Parameters

**Figure 8:**
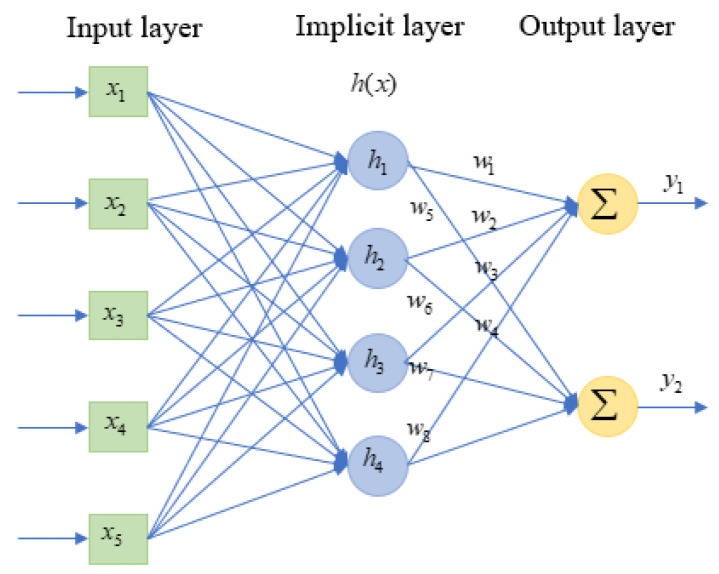
Structure diagram of RBFNN.

The three-layer structure of the RBFNN is shown in Figure 8, including the input layer, hidden layer, and output layer. The input is the data source node. The transformation from the input layer to the hidden layer is achieved using a nonlinear Gaussian basis function, while the transformation from the hidden layer to the output layer is linear. The output of the hidden layer is represented as a quadratic term through the weights.

The choice of the radial basis function in an RBFNN determines its mapping ability. The Gaussian kernel function is the most used radial basis function, as shown in the following equation:

(22) $$h_{j}(t)=\exp(-\frac{{\left\|x\left(t\right)-c_{j}\left(t\right)\right\|}^{2}}{2b_{j}^{2}}),\;j=1,\ldots ,m$$

In Equation (20), $c_{j}\left(t\right)$ and $b_{j}$ represent the center and width of the kernel function, respectively, and are calculated using an optimization method based on an improved k-means clustering algorithm.

The desired trajectory angle θ and angular velocity $\dot{\theta }$ of the PAM are sampled to form a sample set $D=\left\{x_{1},x_{2},\cdots ,x_{m}\right\}$. A random sample from D is selected as the centroid $\mu_{1}$. Additional centroids are selected based on the following criteria. When selecting a new centroid $\mu_{r+1}$, the minimum distance between the samples ($x_{j})$ and the selected centroids ($\mu_{1},\mu_{2}\cdots \mu_{r}$) is calculated as follows:

(23) $$D\left(x_{j}\right)=\arg min{\left\|x_{j}-\mu_{1,2\cdots ,r}\right\|}^{2}$$

The larger the value of $D\left(x_{i}\right)$ is, the higher the probability that the sample will be selected until k centroids are selected is. Next, the distance $d_{ji}={\left\|x_{j}-\mu_{i}\right\|}_{2}$ is calculated from each sample $x_{j}$ to each centroid.

(24) $$d_{ji}={\left\|x_{j}-\mu_{i}\right\|}_{2}$$

The variable $x_{j}$ is assigned to cluster $C_{i}$, corresponding to the nearest centroid. Finally, the centroid of each cluster$\;C_{i}$ is computed to update the original centroids:

(25) $$\mu_{i}=\left(\frac{1}{\left|C_{i}\right|}\right)\cdot \sum x,x\in C_{i}$$

The final centroids $\mu_{1,\cdots ,k}$ are the centers $c_{j}\left(t\right)$ of the kernel functions.

Next, the average distance between each centroid and the other centroids is calculated as the basis for width selection:

(26) $$meanD\left(\mu_{j}\right)=\frac{\sum_{i\neq j}dis\left({\mu_{i},\mu }_{j}\right)}{k-1}$$

For the cluster $C_{j}$ belonging to the same cluster center $\mu_{j}$, the variance and scaling factor are calculated as follows:

(27) $$S_{j}=\frac{1}{size\left(C_{j}\right)}\sum_{x_{i}\in C_{j}}dis{\left(x_{i},\mu_{j}\right)}^{2}$$

(28) $$\varepsilon_{j}=\frac{S_{j}}{\frac{1}{k}\sum_{j=1}^{k}S_{j}}$$

The width corresponding to each center is then given by the following:

(29) $$b_{j}=\varepsilon_{j}\cdot meanD\left(\mu_{j}\right)$$

For a specific approximation error, the distribution of hidden nodes optimized by the improved k-means algorithm and the variance-based width selection scheme will significantly reduce the number of hidden nodes and the complexity of the RBF network structure. The advantages of the optimized allocation scheme for hidden layer nodes can be understood in the following ways: all hidden layer nodes are effective for the approximation task, and unnecessary hidden nodes disappear, thus significantly reducing the number of hidden nodes, especially for high-dimensional input RBFs; each hidden layer node works at its maximum approximation capacity.

#### 3.2.2. Control Law

Equation (21) can be simplified as a static model of PAMs as follows:

(30) $$M\left(\theta \right)\ddot{\theta }+C\left(\theta ,\dot{\theta }\right)\dot{\theta }+G\left(\theta \right)=k_{p}u$$

where θ,$\;\dot{\theta }$, and $\ddot{\theta }$ are the vectors of PAM bending angle, angular velocity, and angular acceleration, respectively, and u is the input air pressure vector. This equation is rewritten as a second-order nonlinear system of bending PAMs.

(31) $$\ddot{\theta }=-\frac{1}{M\left(\theta \right)}\left[C\left(\theta ,\dot{\theta }\right)\dot{\theta }+G\left(\theta \right)\right]+\frac{k_{p}}{M\left(\theta \right)}u$$

State variables are used to represent $x_{1}=\theta$, $x_{2}={\dot{x}}_{1}$ and $y=x_{1}$.

(32) $$\left\{\begin{array}{c} x_{2}={\dot{x}}_{1}\\ {\dot{x}}_{2}=f(x_{1},x_{2})+g(x_{1},x_{2})u\\ y=x_{1} \end{array}\right.$$

where $f\left(\theta ,\dot{\theta }\right)$ and $g\left(\theta ,\dot{\theta }\right)$ are nonlinear functions and $u\in R^{n}$ and $y\in R^{n}$ are the control inputs and outputs of the system, respectively.

The desired tracking trajectory is $y_{d}$; then, the error can be expressed as follows:

(33) $$e=y_{d}-y=y_{d}-x_{1},E=\left(e,\dot{e}\right)$$

Substituting the above equations into the second-order nonlinear system yields the error system:

(34) $$\ddot{e}+k_{p}e+k_{d}=0$$

Let $K={\left(k_{P},k_{d}\right)}^{T}$ so that the roots of polynomial $s^{2}+k_{d}s+k_{p}=0$ are all in the left half of the complex plane. Then when t→∞, $e\left(t\right)\to \infty$ and $\dot{e}\left(t\right)\to \infty$.

The RBFNN is employed to approximate the nonlinear function:

(35) $$f=W^{T}h\left(x\right)+\varepsilon$$

Replacing the nonlinear function in Equation (29) with the RBFNN output gives the control law:

(36) $$\left\{\begin{array}{c} u=\frac{1}{g\left(x\right)}\left[-\overset{̑}{f}\left(x\right)+{\ddot{y}}_{d}+K^{T}E\right]\\ \overset{̑}{f}\left(x\right)={\overset{̑}{W}}^{T}h\left(x\right) \end{array}\right.$$

The constant γ and constant matrix Q are the parameters that need to be set in the experiment. The designed adaptive law for weight updating can be expressed as follows:

(37) $$\dot{\hat{W}}=-\gamma E^{T}PBh\left(x\right)$$

**Figure 9:**
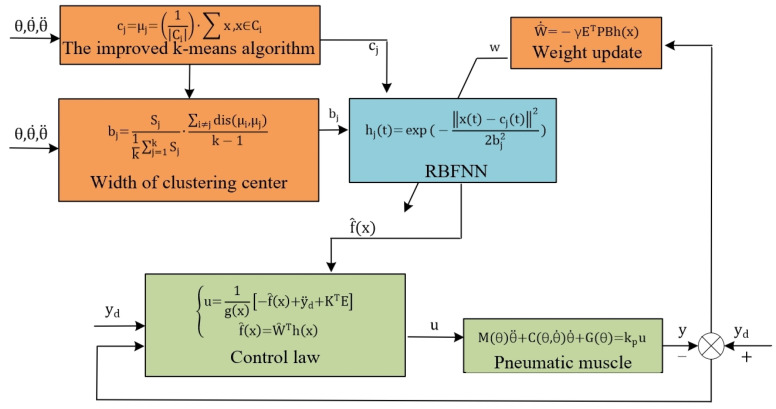
Closed-loop system RBFNN control block diagram.

The RBFNN adaptive control is illustrated in Figure 9. The center $c_{j}$ and width $b_{j}$ of the Gaussian function in the neural network are determined using an improved k-means algorithm, while the weights $\hat{W}$ are updated in real time according to an adaptive law. This allows the neural network to continuously approximate the unknown nonlinear components $\overset{̑}{f}\left(x\right)$ of the PAM dynamic model, ultimately computing the output u based on the control law. Stability analysis can be found in Appendix A.

## 4. Experiments and Results

### 4.1. Experiment Setup

**Figure 10:**
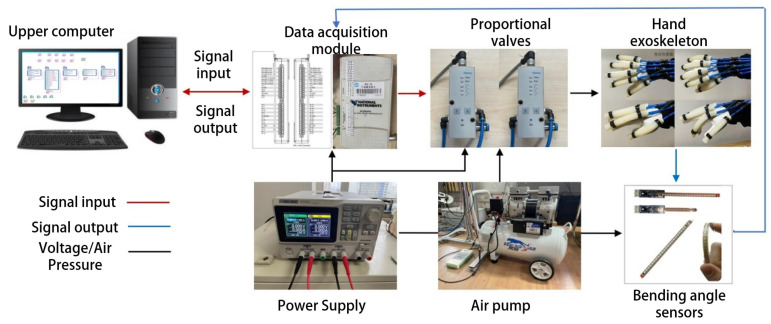
The hand exoskeleton system.

The hand exoskeleton system, as shown in Figure 10, consisted of an upper computer, a data acquisition module, an air pump, proportional valves, bending angle sensors, and the hand exoskeleton. To accommodate varying finger lengths, flexible angle sensors, such as the customizable version of the Sparkfun FLEX4.5, were used. The data acquisition module (Product Model: USB-6210; Company: National Instruments) provided real-time feedback of bending angle signals to the upper computer. The upper computer processed these data and output corresponding voltage signals through the data acquisition module to control the proportional valve. The proportional valve adjusted the air pressure in the PAM cavity according to changes in input voltage, resulting in the PAMs bending to different angles.

We conducted three sets of experiments. The first set involved using finite element simulation software, Abaqus, to analyze the impact of various factors such as wall thickness, length, radius, and cavity shape on the performance of PAMs. Based on the optimal parameters identified from these simulations, PAMs were fabricated, and their bending characteristics were compared between physical experiments and simulations. The second set of experiments focused on controlling a single PAM, comparing the proposed RBFNNO control algorithm with other algorithms to demonstrate its effectiveness. The third set of experiments tested the hand exoskeleton in both passive training mode and active grasping mode. In passive training mode, the PAMs assisted the fingers in moving along a specified trajectory. In active grasping mode, the motion trajectory of the healthy hand was captured using a motion capture system, and the exoskeleton worn on the affected hand was controlled to perform the corresponding grasping task.

**Figure 11:**
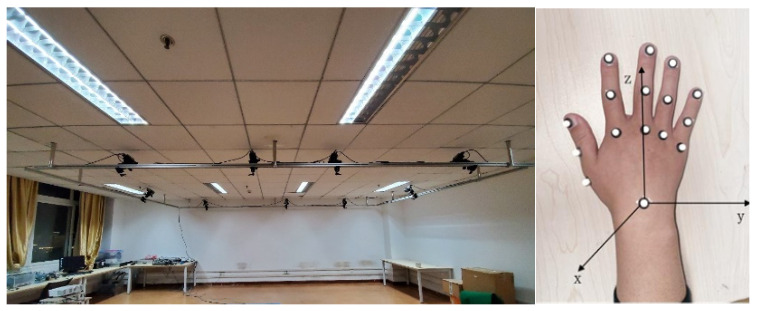
Motion capture system.

To prevent data loss due to occlusion during movement, we selected reflective markers with a diameter of 3 mm, distributing them evenly across the joints of the hand. The placement of the hand markers was shown in Figure 11. Each of the five fingers had three markers attached at the MCP, proximal interphalangeal (PIP), and DIP joints to calculate finger bending angles. A reflective marker was placed on the wrist to serve as the origin of the spatial coordinate system. MATLAB was used to process the coordinate changes of each marked point and convert them into joint angles

### 4.2. Simulation and Physical Testing of PAM

To simulate the flexion and extension movements of fingers, PAMs operate in two modes: flexion and extension. Several factors, including the length of the parallel chamber actuator, cavity shape, eccentric structure of the pneumatic chamber, cavity radius, and helix line, influence the bending deformation effect of these pneumatic muscles. This section explores the factors affecting the bending and extension deformations of PAMs through a simulation analysis.

**Figure 12:**
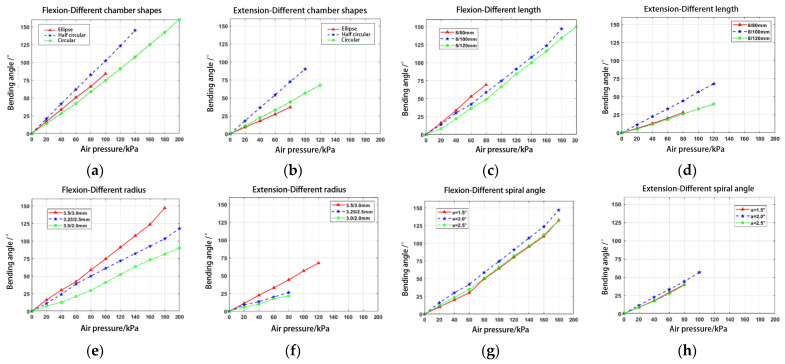
Finite element simulation results: (**a**,**b**) flexion/extension of PAMs with different chamber shapes; (**c**,**d**) flexion/extension of PAMs with different length; (**e**,**f**) flexion/extension of PAMs with different radius; and (**g**,**h**) flexion/extension of PAMs with different spiral angle.

As shown in Figure 12a,b, the best performance is achieved with a semicircular cross-section. However, it is challenging to design a suitable parallel chamber structure, so we opted for a circular cavity. Figure 12c,d indicated that longer PAMs result in greater bending and extension angles. Considering the length of the fingers, we chose 120 mm long PAMs to assist the index, middle, and ring fingers, and 100 mm long PAMs for the other fingers. Figure 12e,f showed that larger cavity radii can enhance bending ability under high pressure. To maintain a sufficient wall thickness and prevent rupture during inflation, we set the radius to 3–3.5 mm based on finger size. Figure 12g,h demonstrated that both larger and smaller spiral angles of the fiber lines reduce bending performance, especially at higher pressures. Therefore, we selected a spiral angle of 2° for the bi-directional winding of the fiber lines.

Based on the analysis of the simulation results, the parameters affecting the bending of the PAM, ranked from largest to smallest influence, are as follows: wall thickness, length, chamber diameter, cavity shape, and center diameter. To achieve an optimally bending PAM, geometric parameters such as longer length, larger chamber diameter, smaller center diameter, and thinner wall thickness are preferred. However, considering actual rehabilitation needs, manufacturing difficulty, durability, large pressure tolerance, and power density, we selected two PAMs with a length of 100/120 mm, a chamber diameter of 3.5/3.0 mm, a center diameter of 8 mm, and a wall thickness of 1.5 mm for physical fabrication.

**Figure 13:**
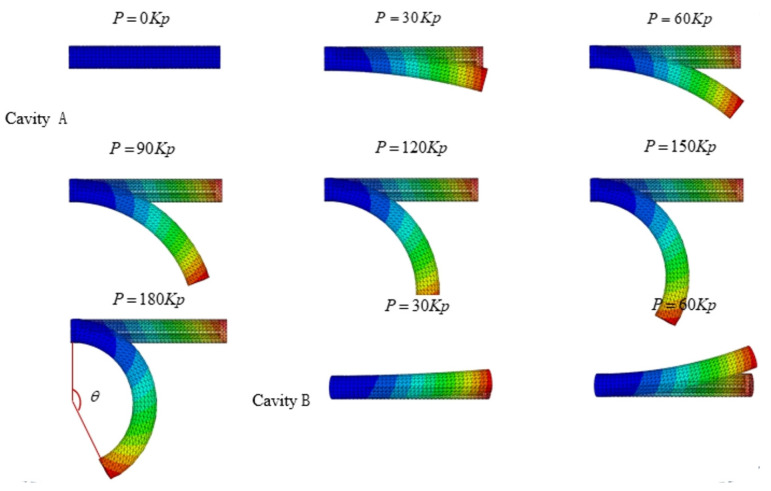
Finite element analysis of flexion and extension.

To verify the effectiveness, we performed a finite element analysis on the flexion and extension movements of the PAMs. The simulation applied a load to one of the parallel chambers, using pressure settings from 0 to 180 kPa in increments of 20 kPa. The displacement contour plots of the PAM in ABAQUS for this state were shown in Figure 13. The first seven images illustrated the application of air pressure from 0 to 180 kPa in Chamber A, corresponding to finger flexion. The last two images showed air pressure applied from 30 kPa to 60 kPa in Chamber B, corresponding to finger extension.

Next, we manufactured the PAM based on the selected parameters. Inflation pressure was incrementally increased by 20 kPa, ranging from 0 kPa to 180 kPa. Once the PAM reached the maximum inflation pressure and stabilized, the steady-state bending angle and fingertip force were measured. Ten steady-state tests were performed at each pressure level. To ensure accuracy, the data were averaged after excluding the maximum and minimum values.

**Figure 14:**
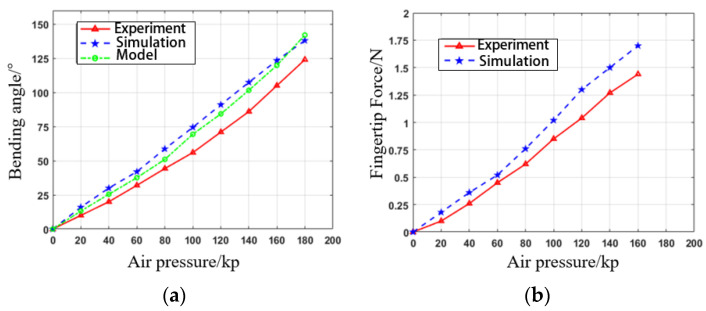
PAM performance testing: (**a**) bending angle; (**b**) fingertip force.

The experimental test results shown in Figure 14 closely match the static model simulations of the PAM. Some errors in the bending angle curve during the actual test were due to the exclusion of the gravity term in the simulation and modeling. The maximum forward bending angle reached 125°, while the reverse bending angle reached 50°. The tests confirmed that the PAM’s bending angles exceeded the normal range for finger bending and extension. Therefore, PAMs can be integrated with soft fabric gloves to assist patients in repetitive grasp training.

### 4.3. PAM Angle Tracking Control

To validate the effectiveness of the proposed control algorithm, both simulations and experimental tests were conducted on the control of a single PAM. The desired trajectory was set to $y_{d}=0.5\sin\pi t+0.5$. The initial state was $x_{1}=0$, $x_{2}=0$. Parameters were set as $Q=\left[\begin{array}{cc} 500 & 0\\ 0 & 500 \end{array}\right]$, $k_{d}=50$, $k_{p}=30$, and the adaptive parameter γ = 1200. A duration of 2000 s was selected for trajectory tracking. The first 20 s (initial state) and the last 20 s (stable tracking) were analyzed separately, with a sampling interval of 0.01 s.

The distribution of RBFNN hidden layer nodes was optimized using the improved k-means algorithm. The number of clustering centers, which corresponded to the hidden layer nodes of the RBFNN, was set to 5, 10, 15, and 20. For each cluster center, a variance-based width optimization algorithm was used to calculate the width of each radial basis function. The initial weights of the network were set to zero and were iteratively updated using an adaptive rate. The parameters $c_{i}$ were determined based on the clustering centers selected by the algorithm.

**Figure 15:**
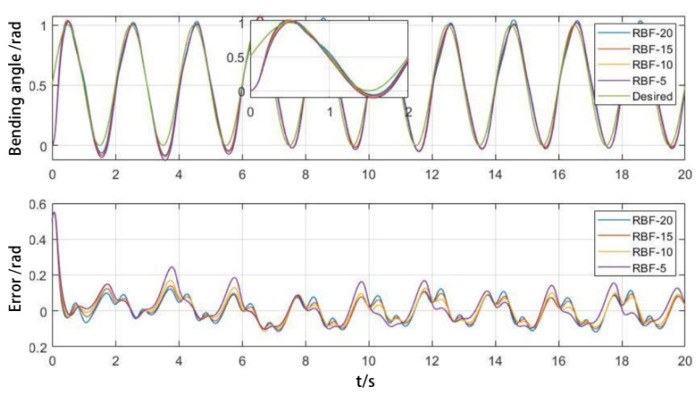
Impact of implicit layer nodes on initial trajectory tracking.

As shown in Figure 15, when tracking the desired trajectory, the RBFNN becomes more complex with an increasing number of hidden layer nodes, resulting in improved tracking of the PAM trajectory. The PAMs start at rest, leading to a significant initial tracking error. Due to the nonlinearity and hysteresis, the tracking error is most pronounced in the inflation deflation transition stage.

**Figure 16:**
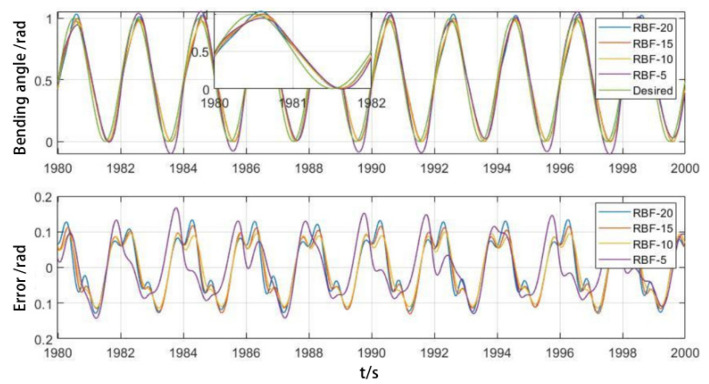
Influence of implicit layer nodes on stable trajectory tracking.

The more hidden layer nodes there are in the initial state, the better the trajectory tracking effect is, and the number of hidden layer nodes accelerates the initial tracking effect. However, as shown in Figure 16, after achieving stable tracking, the error of the RBFNN with 20 hidden layer nodes increases and becomes larger than that of the RBFNN with 10 and 15 hidden layer nodes. The results indicate that reducing the number of hidden layer nodes not only reduces the complexity of the neural network but also improves stability.

**Figure 17:**
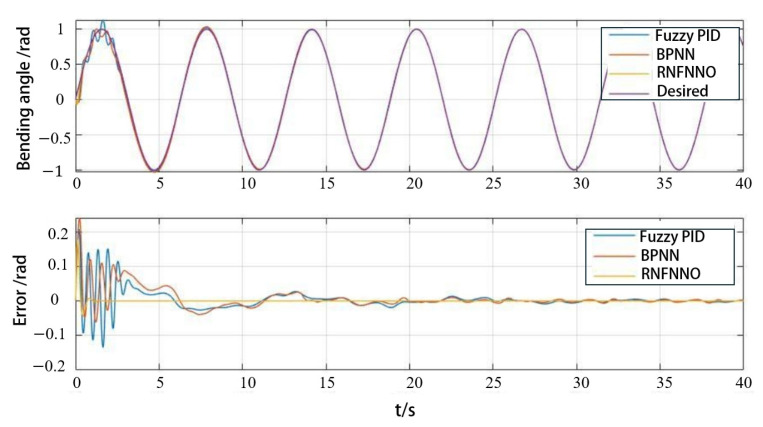
Comparison of different algorithms in PAM angle tracking control.

To verify the advantages of the RBFNNO method, fuzzy PID control and BP neural network control were selected for a comparison analysis. The PAM trajectory tracking is shown in Figure 17. It can be seen that the error convergence is fast, and the control accuracy is high when using the RBFNNO method.

### 4.4. Hand Exoskeleton Control

To validate the role of the hand exoskeleton in aiding rehabilitation training for patients, passive rehabilitation training for each finger was conducted after the patient was fitted with the exoskeleton. Each finger was controlled by individual PAMs with minimal coupling between fingers. The desired trajectory was tracked using the RBFNNO methods.

**Figure 18:**
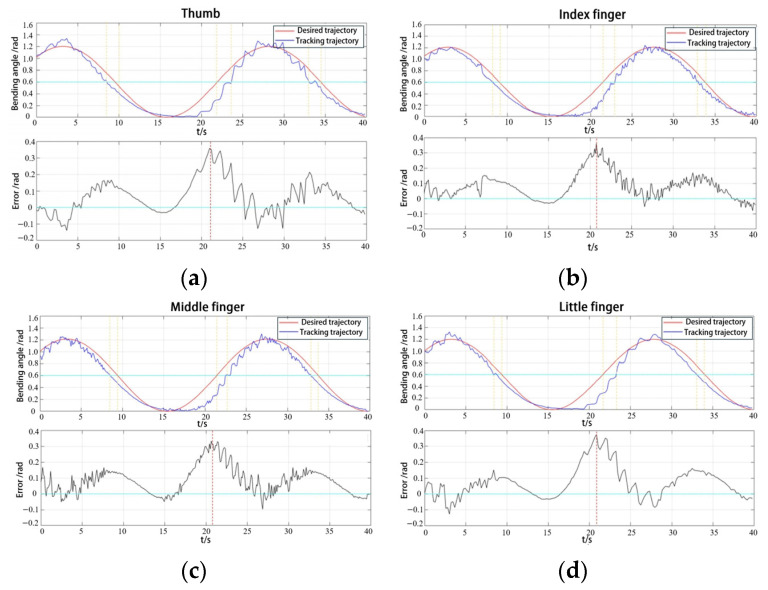
Angle tracking result: (**a**) thumb; (**b**) index finger; (**c**) middle finger; and (**d**) litter finger.

Based on the rehabilitation needs, the maximum bending angle was designed to be 1.2 radians, with one inflation and deflation cycle lasting 25 s for slow rehabilitation. When the angle signal is tracked stably, the angle tracking trajectory and angle trajectory tracking error results for the five fingers are shown in Figure 18.

The maximum error of the five fingers is primarily due to the hysteresis effect and jitter. As shown in Table 3, the mean squared deviation of the thumb is greater than that of the index finger despite having relatively small mean error values. From the Figure 18, it is evident that the hysteresis effect of the thumb is slightly smaller than that of the index finger. For the shorter PAMs, it is generally easier to return to the initial position during the deflation phase, although their jitter and vibration are more pronounced.

In the active grasping mode, the trajectories of the five healthy fingers during object grasping were first collected to serve as the target trajectory for the hand exoskeleton. The subjects were instructed to sit in a relaxed state at the center of the motion capture space and perform full grasping movements using their healthy hand. The hand movement coordinates were recorded from the start of grasping, through object manipulation, to the completion of the grasping action.

**Figure 19:**
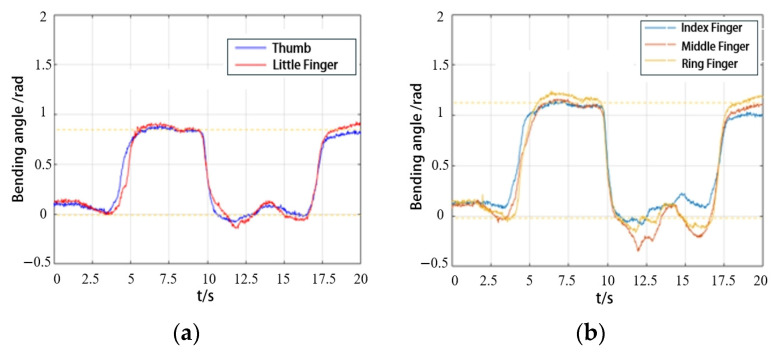
The angle trajectory of grasping objects with healthy hands: (**a**) the thumb and little finger; (**b**) the index finger, middle finger, and ring finger.

Figure 19a showed the angle changes of the thumb and little finger during gripping actions in a healthy hand. Figure 19b displayed the angle changes of the remaining fingers during the same action. The extracted finger trajectories from the healthy hand served as the desired trajectories for the exoskeleton.

**Figure 20:**
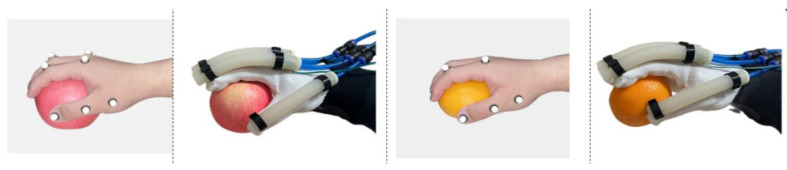
Grasp an apple and an orange with an exoskeleton.

**Figure 21:**
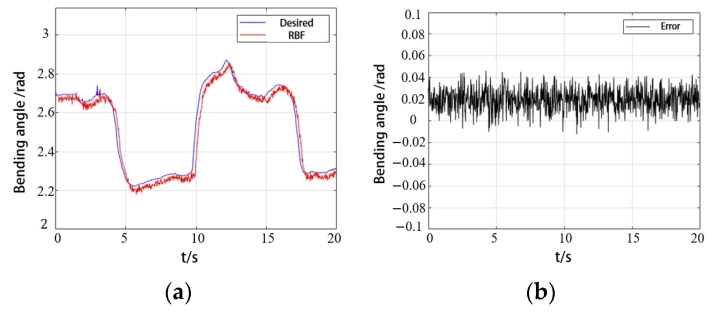
Index finger trajectory tracking in grasping: (**a**) bending angle; (**b**) error.

The exoskeleton assisted the affected hand in completing grasping movements according to the trajectories recorded from the healthy hand. Figure 20 showed the gripping action of a healthy hand and the affected hand wearing the exoskeleton while grasping the same object. Figure 21 illustrated the trajectory changes of the affected hand’s index finger during the gripping process along with the error compared to the healthy hand’s index finger trajectory. As demonstrated in Figure 20 and Figure 21, the hand rehabilitation exoskeleton effectively aids patients with muscle weakness in grasping different objects.

## 5. Discussion

By analyzing the mechanism of finger joint movement and the rehabilitation needs of the hand, a parallel chamber PAM with bidirectional bending capabilities was designed to assist patients in finger flexion and extension movements. The bending performance of the PAM with different structural parameters was compared using a finite element analysis, and the structural parameters were optimized based on actual rehabilitation needs. The mold design was carried out in SOLIDWORKS, and the mold was fabricated using 3D printing technology.

Compared to rigid hand rehabilitation exoskeletons [26] and motor-driven soft hand rehabilitation exoskeletons [27], the designed actuator is lighter and soft, with a single PAM weighing only 25 g, making it more comfortable to wear. Compared to existing soft hand exoskeletons driven by PAMs, our developed exoskeleton features an improved structure and algorithm, resulting in enhanced performance and higher precision. The bending angle of the PAM designed by Li et al. [28] for hand rehabilitation is only around 0 to 70°, which is much smaller than the −50° to 125° range of our developed PAM. The fingertip force of the rehabilitation glove developed by Han et al. [12] is approximately 0.40 N to 0.50 N, which is lower than the fingertip force of 1.25 N of the exoskeleton we developed. Additionally, many PAM-driven hand rehabilitation exoskeletons [15] only assist with finger bending and cannot help patients with muscle weakness extend their fingers for repeated grasping. The bidirectional bending capability of our PAM better supports finger extension and enables patients to complete multiple grasping tasks in daily life.

We derived a dynamic model of the PAM by substituting the reduced-order forms of strain energy, generalized force, kinetic energy, and gravitational potential energy into the Lagrange equation. Given the requirement for precise position control of PAMs during rehabilitation training, an optimized radial basis function neural network adaptive control algorithm was developed, focusing on parameter and hidden layer node optimization. The algorithm utilizes a k-means clustering algorithm to determine the centers of the radial basis functions and optimizes their widths based on sample distribution, thereby accelerating network convergence and enhancing the position control accuracy. This approach offers higher accuracy and greater stability compared to control methods such as PID, fuzzy logic, and unoptimized neural networks used in other hand rehabilitation exoskeletons. For example, the PID control method used by Tejada et al. [29] can only bend the pneumatic muscles to 90% of the maximum expected angle. Haghshenas et al. [30] reported an average error of 12.4° in the movement of the index finger during bilateral therapy.

We designed both a passive training mode and an active grasping mode for the hand exoskeleton. In the early stages of rehabilitation, patients undergo passive flexion and extension training for individual fingers or the entire hand. In the later stages, grasping rehabilitation training is implemented using joint trajectory mapping between the healthy and affected hands to enhance hand muscle strength during active rehabilitation. The combination of these modes provides more effective support for patients in completing rehabilitation tasks and daily activities.

Our research currently has some shortcomings. Single fingers can only flex and extend, lacking the ability for independent control of multiple joints in one finger or for abduction/adduction. The thumb’s multi-degree-of-freedom motion requires more advanced PAMs. We plan to improve the design of PAMs and the exoskeleton to better match hand movement characteristics. Additionally, using motion capture systems or multiple bending sensors may make the exoskeleton cumbersome and difficult to use, so we aim to investigate PAMs with embedded sensors to enhance wearability. Since hand sizes vary among users, the required sizes for the exoskeleton and PAMs will differ as well. To improve usability and simplify manufacturing, we need to develop an automated design tool that customizes PAM and exoskeleton parameters based on individual hand sizes.

In terms of control, we also need to reduce response time and enhance human–machine interaction. The current control method has significant errors and delays during the inflation and deflation phases, which require improvement. In the future, we could maintain a certain pressure in one chamber while inflating the other. This control method, resembling antagonistic muscle pairs, would allow us to adjust the stiffness of the PAM. Additionally, we hope to incorporate physiological signals, such as electromyographic (EMG) signals, to monitor the patient’s movement capabilities in real time. This would enable the exoskeleton to automatically switch between active and passive grasping training.

## 6. Conclusions

This paper explores the design, modeling, and control methods of bidirectional bendable PAMs for the soft hand rehabilitation exoskeleton. A PAM dynamic model based on the Lagrangian method is established, and an adaptive control algorithm using an RBFNN with parameter and node optimization is designed to achieve accurate position control. The integration of PAMs with fabric gloves results in a softer and more wearable hand rehabilitation exoskeleton compared to motor-driven hand exoskeletons, better aligning with the rehabilitation needs of stroke patients. We have developed both passive training and active grasping modes to enhance patients’ grasping ability and increase their motivation for rehabilitation. Next, we plan to enhance the functionality of exoskeletons by improving the performance of PAM and integrating physiological signals such as EMG. We plan to collaborate with hospitals in the future to conduct more experiments involving patient participation. This involvement will better validate the effectiveness of the rehabilitation exoskeleton across various rehabilitation modes.

## Figures and Tables

**Table 1:** PAM parameters.

Parameters	Numerical Value
Drive length/mm	100/120
Drive radius/mm	8
Inner diameter of cavity A/mm	3.5
Inner diameter of cavity B/mm	3.0
Distance of cavity A center deviation from drive center/mm	3.5
Distance of cavity B center deviation from drive center/mm	3.0
Number of spiral wire windings N	34/42
Winding pitch/mm	3
Drive mass/g	25/31

**Table 2:** Actual range of motion of PAMs.

Finger	Flexion Angle	Extension Angle
Thumb	0–89°	0–12°
Index finger	0–118°	0–21°
Middle finger	0–120°	0–18°
Ring finger	0–119°	0–20°
Little finger	0–91°	0–11°

**Table 3:** Trajectory tracking error for five fingers.

Finger	Maximum Error/Rad	Mean Error/Rad	Mean Square Error/Rad
Thumb	0.3308	0.0651	0.1219
Index finger	0.3596	0.0788	0.1141
Middle finger	0.3291	0.0815	0.1183
Ring finger	0.3194	0.0803	0.1179
Little finger	0.3517	0.0705	0.1217

## Data Availability

The data and code of the current study can be obtained from the corresponding author upon reasonable request.

## Appendix A. Stability Analysis

Substituting Equation (36) into Equation (32) yields the expression for the closed-loop system as follows:

(A1) $$\ddot{e}=-K^{T}+\left[\hat{f}\left(x\right)-f\left(x\right)\right]$$

Due to $\Lambda =\left[\begin{array}{cc} 0 & 1\\ {-k}_{p} & {-k}_{d} \end{array}\right]$, $B=\left[\begin{array}{c} 0\\ 1 \end{array}\right]$, the above equation can be rewritten as

(A2) $$\dot{E\;}=\Lambda E+B\left[\hat{f}\left(x\right)-f\left(x\right)\right]$$

The optimal weight is given by

(A3) $$\;W^{*}=argmin\left[sup\left|\hat{f}\left(x\right)-f\left(x\right)\right|\right]$$

The model approximation error is defined as follows:

(A4) $$\omega =\hat{f}\left(x\left|W^{*}\right.\right)-f\left(x\right)$$

Equation (36) can be written as follows:

(A5) $$\dot{E\;}=\Lambda E+B\left\{\left[\hat{f}\left(\left.x\right|\right)-\hat{f}\left(x\left|W^{*}\right.\right)\right]+\omega \right\}$$

Substituting $\overset{̑}{f}\left(x\right)={\overset{̑}{W}}^{T}h\left(x\right)$ into the above equation yields the following:

(A6) $$\dot{E\;}=\Lambda E+B\left[{\overset{̑}{(W}-W^{*})}^{T}h\left(x\right)+\omega \right]$$

The Lyapunov function is designed as follows:

(A7) $$V=\frac{1}{2}E^{T}PE+\frac{1}{2\gamma }{\left(\hat{W}-W^{*}\right)}^{T}\left(\hat{W}-W^{*}\right)$$

where γ is a constant and the matrix P is symmetric positive definite and satisfies the following Lyapunov equation:

(A8) $$\Lambda^{T}P+P\Lambda =-Q$$

where Q>0; let $V_{1},V_{2}$ be

(A9) $$V_{1}=\frac{1}{2}E^{T}PE$$

(A10) $$V_{2}=\frac{1}{2\gamma }{\left(\hat{W}-W^{*}\right)}^{T}\left(\hat{W}-W^{*}\right)$$

Then, differentiating $V_{1}$ and $V_{2}$, we derive the following:

(A11) $$\dot{V}=-\frac{1}{2}E^{T}QE+E^{T}PB\omega +\frac{1}{\gamma }(\hat{W}-W^{*}{)}^{T}\left[\dot{\hat{W}}+\gamma E^{T}PBh\left(\theta \right)\right]$$

Substituting Equation (34) into the above equation, we obtain the following:

(A12) $$\dot{V}=-\frac{1}{2}E^{T}QE+E^{T}PB\omega$$

Since $-\frac{1}{2}E^{T}QE\leq 0$, the RBFNN can be designed such that the approximation error w is sufficiently small, making $\dot{V}\leq 0$.

‖B‖ = 1 and $\left|\omega \right|\leq \omega_{max}$, then $\dot{V}\leq =-\frac{1}{2}\left\|E\right\|\left[\lambda_{min}\left(Q\right)\left\|E\right\|-2\omega_{max}\lambda_{max}\left(P\right)\right]$, where $\lambda_{min}$ is the minimum eigenvalue of matrix Q and $\lambda_{max}$ is the maximum eigenvalue of matrix P.

Since $\dot{V}=0$, if and only if $\left\|E\right\|=\frac{2\omega_{max}\lambda_{max}\left(P\right)}{\lambda_{min}\left(Q\right)}$, as t→∞, $\left\|E\right\|\to \frac{2\omega_{max}\lambda_{max}\left(P\right)}{\lambda_{min}\left(Q\right)}$, and the convergence rate of the system depends on $\lambda_{min}$.

Given V≥0 and $\dot{V}\leq 0$, when t→∞, V is bounded, implying that $\tilde{W}$ is also bounded. In summary, the system is stable.

## Appendix B. Variable Definition

**Table A1:** Definition and explanation of variables.

Variable	Definition	Variable	Definition
R	Bending radius of PAM	$e_{1}$	Eccentricity of cavity A
$e_{2}$	Eccentricity of cavity B	θ	Bending angle of PAM
$r_{10}$	Original radius of cavity A	$r_{20}$	Original radius of cavity B
$L_{1}$	Length of the central axe of cavity A	$L_{2}$	length of the central axe of cavity B
$C_{i0}$	Yeoh model material parameter.	$\lambda_{1}$	Axial stretch ratios
$\lambda_{2}$	Radial stretch ratios	$\lambda_{3}$	Circumferential stretch ratios
$L_{0}$	Initial length of the central axis	$R_{0}$	Initial radius of the PAM
$t_{10}$ $/t_{30}$	The thickness of the front and rear sides of cavity A		
$t_{20}$ $/t_{40}$	Thickness of the left and right sides of cavity A		
$t_{1}$ $/t_{2}$ $/t_{3}$ $/t_{4}$	$t_{10}$$/t_{20}$$/t_{30}$$/t_{40}$ after inflating	β	The variation of the thickness along the bottom to the top
$\rho_{w}$	Strain energy density	$E_{g}$	Gravitational potential energy
m	PAM mass	g	Gravitational acceleration
$E_{w}$	Strain energy	$E_{k}$	Kinetic energy
s	The arc length between the point on the PAM and the origin		
$f_{p}$	Generalized force	$V_{h}(\theta )$	Volume of the cavity A
u	Air pressure	$L_{f}$	Lagrangian function
$k_{1}$ $/k_{2}$ $/k_{3}$ $/k_{p}$	Coefficients used to simplify equations	ξ	The coefficient for the damping characteristics and resistance
$h_{j}(t)$	Gaussian kernel function	x(t)	Input of RBFNN
$c_{j}(t)$	The center of the kernel function	$b_{j}$	The width of the kernel function
W	Weight of RBFNN	$\hat{W}$	Estimated value of W
D	Sample set	$\mu_{i}$	Clustering center
$x_{j}$	A sample in D	$C_{i}$	$\mathrm{Cluster}\;\mathrm{belong}\;\mathrm{to}\;\mu_{i}$
$d_{ji}$	$\mathrm{Distance}\;\mathrm{from}\;x_{j}$ $\;\mathrm{To}\;\mu_{i}$	$S_{j}$	The variance
$\varepsilon_{j}$	Scaling factor	y	Actual trajectory
$y_{d}$	Desired trajectory	$\overset{̑}{f}\left(x\right)$	Estimated value of f(x)
